# A “Cold in the Head”

**Published:** 1891-03

**Authors:** 


### A "COLD IN THE HEAD."

It is probable, that not more than one in ten supposed colds have any connection with the closing of the pores. Most, if not all, of the irritation in the nasal passages, the inflammation of the mucous surfaces, not only of the nasal passages, but of the throat, &c., with the sores about the nose and on the lips, usually regarded as "cold sores," have their origin in a deranged state of the stomach, the inner surface of this organ having a similar appearance. As a result of improper dietetic habits—taking food very difficult of digestion, too much of ordinary food, or at improper times, and eating so rapidly that it is not half masticated—some have a continuous "head cold," and are unable to breathe with the mouth closed, thus inducing additional disease. The appropriate treatment of such supposed colds, &c., is the adoption of simple habits, careful dieting, making the grains and fruits more than usually prominent, eating flesh very sparingly, if at all, and no pork. These supposed colds have led many persons to take undue care of the head, in contrast with the feet, which demand a great deal more attention as the means of warding off such dreaded evils. Another doctor says there is no doubt that many colds come through the feet. Thin soled shoes, or thick soles, standing on ice or snow, or cold wood, until the sole attains the same degree of cold as that on which it rests; then cold feet, cold legs, cold abdomen, cold lungs, cold in the throat, and in the head.

